# Exploring factors shaping employment outcomes of people with disabilities through the PEOP model: a scoping review

**DOI:** 10.3389/fresc.2026.1725152

**Published:** 2026-04-28

**Authors:** Huiling Hu, Peter H. F. Ng, Karen P. Y. Liu, Andy S. K. Cheng

**Affiliations:** 1 Department of Rehabilitation Sciences, The Hong Kong Polytechnic University, Hung Hom, Hong Kong SAR, China; 2 Department of Computing, The Hong Kong Polytechnic University, Hung Hom, Hong Kong SAR, China; 3 Department of Health and Physical Education, The Education University of Hong Kong, Taipo, Hong Kong SAR, China

**Keywords:** disabled persons, employment, PEOP model, person-environment-occupation-performance model, work

## Abstract

**Introduction:**

Employment is a critical dimension of social participation for individuals with disabilities, yet persistent barriers restrict equitable workforce engagement.

**Objective:**

This scoping review applied the Person-Environment-Occupation-Performance (PEOP) model to systematically examine factors influencing employment among people with disabilities.

**Methods:**

Guided by established scoping review methodological frameworks and reported in accordance with PRISMA-ScR, a comprehensive search was conducted across four major databases (Embase, PubMed, CNKI, and PubScholar) to identify relevant studies published between January 2015 and January 2025. After screening 6,240 records, 20 articles met the inclusion criteria. Data were extracted and synthesized using a theory-informed thematic analysis based on the PEOP model.

**Results:**

The included studies were categorized into the Person (*n* = 13), Environment (*n* = 11), and Occupation (*n* = 11) domains. Person-level factors emphasized motivation, self-efficacy, and demographic characteristics such as disability type, age, and gender. Environmental factors included workplace accommodations, social support, and transportation accessibility. Occupational factors focused on vocational rehabilitation programs and job modifications.

**Conclusion:**

Findings highlight the complex and interrelated influences of personal, environmental, and occupational factors on employment for people with disabilities. Future research should expand to underrepresented regions and populations and develop culturally appropriate, evidence-based interventions to promote inclusive employment.

**Systematic Review Registration:**

https://doi.org/10.17605/OSF.IO/FD8GT

## 1 Introduction

People with disabilities (PWD) experience notably lower employment rates than their peers without disabilities. According to data from the Organization for Economic Co-operation and Development (OECD), which comprises 38 member countries, indicate that PWD are approximately 40% less likely to be employed than those without disabilities (1). The persistent employment gap over the past decade highlights global challenges PWD face in the labor market.

Even minimal employment is associated with improved physical and mental health outcomes and reduced healthcare costs for PWD (2–4). Various policies, such as employment quotas and workplace accommodations, have been implemented to promote inclusion. For instance, China requires that at least 1.5% of employees in a company be PWD (5), while U.S. federal agencies must ensure that 2% of their workforce comprises individuals with targeted disabilities (6). Beyond quotas, OECD countries have implemented disability-related policies, including accessible infrastructure, vocational assessments, workplace accommodations, and employer incentives (7). However, in both OECD countries and China, weak enforcement, limited employer awareness, financial constraints, and a shortage of vocational rehabilitation professionals continue to undermine policy effectiveness (7, 8). These systemic limitations may weaken worker motivation and increase the likelihood of early employment termination (9). These persistent implementation gaps suggest that policy efforts alone are insufficient to address the complexity of disability employment. Employment barriers for PWD remain multifaceted and context-dependent. In Europe and North America, PWD face legal complexities, discrimination, and obstacles such as low education levels, unfavorable job markets, and limited vocational training opportunities, particularly for those with intellectual disabilities and co-occurring mental health conditions (10–12). In China, inadequate access to employment guidance and inequitable treatment by enterprises remain common (13). More broadly, additional factors such as age, race, and family support compound these employment challenges (14).

A structured theoretical framework is needed to understand how these diverse factors shape employment outcomes. Although previous reviews have examined factors influencing the employment of PWD, none has systematically synthesized this evidence within a rehabilitation framework (15–17). Given the multidimensional nature of employment barriers, a model that integrates different perspectives is essential for organizing evidence and informing intervention design. Occupational therapy models provide such a foundation by supporting targeted, systematic, and evidence-based assessment and intervention (18–21). Their importance is further reflected in research evaluation standards of the National Institutes of Health (NIH), professional competency standards of the American Occupational Therapy Association (AOTA), and educational requirements of the Accreditation Council for Occupational Therapy Education (ACOTE) (22–24). Among these, the Person–Environment–Occupation–Performance (PEOP) model is a prominent framework. Initially developed in 1985 and formally published in 1991, it has undergone four editions, with the latest published in 2015 (25–27). The PEOP model aligns closely with the Occupational Therapy Practice Framework, Fourth Edition (OTPF-4), and the International Classification of Functioning, Disability, and Health (ICF), bridging biomedical and sociocultural perspectives (18, 28). It conceptualizes performance as the outcome of interactions among three key dimensions: person, environment, and occupation (26, 29). In the context of this scoping review, performance refers not only to obtaining employment, but also to sustaining employment and carrying out work roles and tasks through the dynamic interaction of personal capacities, environmental conditions, and occupational demands. Consistent with the focus of this review, employment-related performance includes job acquisition, job retention, and participation in work roles within specific social, physical, and organizational contexts. The model's distinctive inclusion of narrative further emphasizes the importance of lived experiences, values, and goals in shaping occupational performance (29). This contextualized perspective supports client-centered analysis by recognizing that employment outcomes are shaped not only by individual characteristics, but also by how PWD experience and respond to their work environments and occupational demands (18).

Since its fourth edition in 2015, the PEOP model has gained increasing recognition in rehabilitation research and practice, making this an opportune time to evaluate its application in understanding disability employment. To address the identified gap, this scoping review applies the fourth edition of the PEOP framework to investigate the question: What factors influence the employment outcomes of PWD? This review aims to deepen understanding of the factors shaping employment outcomes for people with disabilities through the PEOP framework and to generate insights for vocational rehabilitation, workplace inclusion, and policy development.

## 2 Methods

This scoping review was guided by established methodological frameworks for scoping reviews, including the framework of Arksey and O'Malley, subsequent methodological refinements, and the Joanna Briggs Institute guidance for scoping reviews (30–32). The review is reported in accordance with the Preferred Reporting Items for Systematic Reviews and Meta-Analyses extension for Scoping Reviews (PRISMA-ScR) (33) and registered on OSF (Registration DOI: https://doi.org/10.17605/OSF.IO/FD8GT). The process included database selection, formulation of search strategies, the establishment of inclusion criteria, study selection, data extraction, and analysis.

### 2.1 Database selection and search strategy

The first author (HH) and an experienced health science librarian developed the keyword search protocol. Two researchers (HH, KL) independently conducted comprehensive searches in Embase, PubMed, CNKI, and PubScholar to identify relevant studies published between January 2015 and January 2025. Boolean operators, such as “AND” and “OR,” were also utilized in the search strategy (Supplementary Table 1) to obtain the best information between the keywords. All keywords in the two domains were combined in the following way: (“Work” OR “Employment” OR “Occupations” OR “Career Choice” OR “Job Security” OR “Job”) AND (“People with disabilities” OR “Disabled persons” OR “Disabled people” OR “Persons with disabilities”). Search terms were tailored to each database. To ensure completeness, the search was conducted in two rounds; the initial search covered January 2015 to May 2024, followed by an updated search including studies published from June 2024 to January 2025 (34).

### 2.2 Study criteria

Based on this review's research background and objectives, three researchers (HH, PN, KL) collaboratively discussed the inclusion and exclusion criteria (Table 1). It excluded studies involving participants who were not clearly defined as having a disability and a mixture of participants with and without disabilities, where data about PWD were not reported separately. Studies were excluded if participants were recorded as out of working age (16–65). Any form of paid employment was eligible for inclusion in this review, such as competitive employment, supported employment, integrated employment, full-time and part-time jobs, and other types. Studies that were deemed unrelated were discarded, and the full text of the remaining studies was reviewed.

**Table 1:** Eligibility criteria.

Eligibility criterion	Inclusion	Exclusion
Study design	Quantitative and qualitative studies including RCT, non-randomized controlled trials, cross-sectional studies, cohort studies.	Case reports, brief reports/series, editorials, opinion pieces, reviews, systematic reviews, grey literature, policy documents, news, or books.
Date of publication	Recent 10 years from January 2015 to January 2025	Before 2015
Language and accessibility	English or Chinese with full-text available	Neither in English nor Chinese or no full-text available
Study population	People with disabilities in employment age (16-65 years old)	Younger than 16 and older than 65 years old
		Reported as dysfunction, diseases, or work injury without explicitly identifying participants as people with disabilities.
Study outcome	Studies that report employment status outcomes, such as: employment rate, job acquisition, job retention or loss.	Studies that focus on non-employment status outcomes, such as: psychological scales scores related to job satisfaction.

### 2.3 Study selection

**Figure 1:**
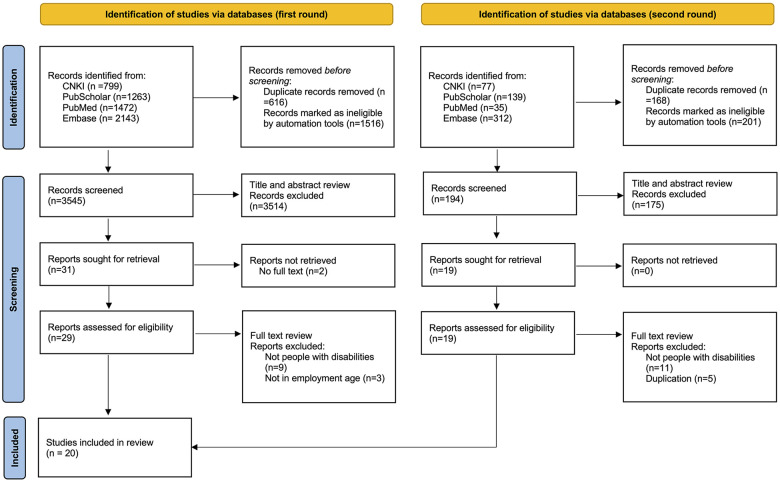
Search and selection process flow diagram.

After implementing the previous stages, the selection process was performed. Using the platform Covidence, two researchers (HH, PN) independently reviewed each title and the abstract to determine which articles merited a full review. The two times of data collected from the databases were as follows: 1,472 and 35 records from PubMed, 2,143 and 312 records from Embase, 799 and 77 records from CNKI, and 1,263 and 139 records from PubScholar. A total of 5,677 and 563 original articles were found. 616 and 168 duplicated records, and 1,516 and 201 ineligible records had been removed. By examining the titles and abstracts of the articles, it was determined that 3,514 and 175 entries did not meet the inclusion requirements and were thus eliminated. The remaining 29 and 19 items were retrieved and evaluated; the full texts of 2 were not found. 12 and 16 records were excluded due to conflicts with the research's goal. Finally, 20 papers (1 in Chinese and 19 in English) were chosen to analyze the complete review. A PRISMA flow diagram (Figure 1) was adopted to illustrate the search and selection process. In this process, when necessary, another researcher (KL) resolved the disagreement regarding the eligibility of the documents. Regular discussions among research team members were conducted to monitor the screening process's progress.

### 2.4 Data extraction

Two researchers (HH, PN) worked independently on data extraction. The data extraction included the following: title, leading author, year of publication, study design, participants, outcome measures, and results (e.g., key findings of barriers and facilitators for employment of PWD) using a standardized data collection form.

### 2.5 Data synthesis and analysis

After data extraction, a theory-informed thematic analysis using the PEOP model was conducted to synthesize the data (35). The analysis followed Braun and Clarke's six-phase framework for thematic analysis (36). Participants were grouped into different narrative levels: Individual, Organization, and Population. Reported findings were then coded into the Person, Environment, and Occupation domains according to the primary content of each factor. Because individual studies often reported findings spanning more than one PEOP domain, coding was non-mutually exclusive. Two researchers (HH, PN) conducted the data synthesis and analysis independently, and the coding was validated by another researcher (KL).

## 3 Results

### 3.1 General characteristics of the studies

The general characteristics of the included studies are presented in Supplementary Table 2. The oldest studies were published in 2015 (14), and the most recent study was published in 2025 (37). Two studies were randomized controlled trials (38, 39), and four were qualitative studies using interview or focus group methods (40–43). Geographically, nine studies were conducted in North America (14, 37, 38, 41, 44–48), nine studies were carried out in Europe (40, 42, 43, 49–54), and two studies were conducted in Asia (39, 55).

Persons with psychiatric or intellectual disabilities were involved in 9 studies (37–39, 42, 44–46, 51, 52). Five studies aimed at people with physical disability (37, 40, 41, 43, 47), while another six studies explored the possible employment factors for PWD from a general perspective (14, 49, 50, 53–55).

### 3.2 Factors affecting the employment of PWD

Twenty included studies reflected the diversity of client narratives within the PEOP framework: four studies were conducted at the individual level, eight at the organization level, and eight at the population level. The employment-related factors identified in these studies were then coded into the Person, Environment, and Occupation domains of the PEOP model (Table 2). Because the coding framework allowed overlap across domains, a single study could contribute to more than one domain. Accordingly, the totals across domains exceed the 20 included studies. The Person domain was the most frequently represented, with 13 studies identifying person-related factors as influencing employment outcomes, followed by the Environment domain (*n* = 11) and the Occupation domain (*n* = 11). Five studies addressed all three domains, indicating that some studies provided a more comprehensive account of the interacting factors shaping employment outcomes.

**Table 2:** Factors affecting the employment of PWD in the PEOP model perspectives.

Type of client	Person (13)	Environment (11)	Occupation (11)
Individual (4) Organization (8) Population (8)	Cognition and memory (3) Character factors (2) Daily living ability (2) Advanced skills and labor ability (5) Motivation (3) Self-esteem (2) Self-efficacy (2) Disability type (2) Disability level or severity (2) Age (4) Race or ethnicity (2) Native English speakers (1) Place of birth (1) Identity of veteran (1) Marital status (1) Comorbidity factors (1) General health (3) Education experience (5) Work experience (4) Social activities participation history (3) Arrest record (1)	Interpersonal relationships (2) Workplace accessibilities and accommodation (3) Assistance from coworker (2) Employers’ attitude and support (3) Stigma and discrimination in workplace (2) Parental expectation and support (2) Family economy (1) Household and caregiving issue (3) Labor market challenges (2) Transportation (4) Social support (2) Reform of policy and laws (1) Inclusion and integration (2)	Vocational rehabilitation intervention (10): supported employment (2), vocational training programs (5), counseling (1), job search strategies (1), working alliance (1), career awareness training (1) Adjustment of work (3): flexible work schedules (2), modified job duties (1), workload (1) Adaptive Gaming (1)

Thirteen studies examined factors related to the Person (14, 38, 40–42, 44–49, 51, 55). Four studies reported that motivation, self-efficacy, and self-esteem facilitate job seeking, whereas health concerns and financial disincentives reduce motivation (40, 44, 46, 49). Eleven studies highlighted the impact of demographic variables such as disability severity, age, and race on employment prospects (14, 41, 42, 44–49, 51, 55). Cognitive deficits and poor prospective memory were found to limit employment opportunities (38, 44), while youth with stronger comprehension skills demonstrated more favorable outcomes (14). Employment can also be hindered by personal characteristics such as reluctance to disclose needs or lack of assertiveness in the workplace (40). In contrast, maintaining an open mindset and proactively seeking support were associated with better workplace integration (41). Advanced skills, including self-advocacy and communication, further enhanced employability (41, 46, 49, 55).

Eleven studies identified Environmental factors shaping employment outcomes (14, 37, 40–44, 46, 49, 54, 55). These included workplace accommodations, interpersonal relationships, and support from employers and coworkers (40, 42, 46). Broader contextual elements, such as social support, transportation access, and family expectations, also played critical roles (14, 41, 43, 46). Two studies additionally emphasized the importance of inclusion and integration within the workplace (42, 44).

Eleven studies explored factors of Occupation, mainly underscoring the significance of vocational rehabilitation interventions and work adjustment (14, 37, 39, 40, 42, 44, 46, 48, 50, 52, 53). Supported employment programs and vocational training significantly improve job and income outcomes, while flexible work schedules and job modifications aid in retention (37, 42, 46). Conversely, heavy workloads hinder sustained employment (42).

## 4 Discussion

Guided by the PEOP model, this review synthesized and categorized factors influencing employment outcomes among PWD. The analysis focused on individuals explicitly identified as having disabilities, rather than those with temporary functional limitations or uncertain recovery trajectories, to provide targeted insights into their sustained employment challenges. The ten-year review period (2015–2025) was selected to reflect the most recent decade of research and policy developments addressing disability employment, aligned with the publication of the fourth edition of the PEOP model and a renewed global emphasis on inclusive work participation from the International Labor Organization (ILO) and the United Nations Economic and Social Commission for Asia and the Pacific (UN ESCAP) in 2015 (18, 56, 57). The following section discusses how the identified factors interact to influence employment within the PEOP framework.

### 4.1 Type of clients

A key strength of the latest PEOP model (29) is the inclusion of narrative, which emphasizes how different types of clients interpret and attribute meaning to their experiences, anchoring findings in personal histories, perspectives, and values that influence decision-making and goal setting (18). Across the 20 included studies, four were conducted at the individual level, eight at the organization level, and another eight at the population level.

Studies involving participants at the individual level (*n* = 4) examined the personal experiences of PWD, such as young adults with chronic physical disabilities or women with multiple sclerosis (40, 43). Although few in number, these studies generated more than half of the identified factors across the Person, Environment, and Occupation domains. This suggests that individual-level research provides nuanced insights into how personal attributes, environmental interactions, and occupational contexts intersect to shape employment outcomes. Studies at the organization level (*n* = 8) focused on participants embedded in specific vocational rehabilitation programs or institutional contexts. Examples include the Supported Employment (SE) program in Canada, Project SEARCH in the United Kingdom, and the Integrated Supported Employment (ISE) program in China, as well as samples drawn from rehabilitation hospitals (39, 44, 52). These studies primarily emphasized Occupation-related factors, such as vocational rehabilitation interventions, supported employment services, and job adjustments, illustrating how organizational practices influence work participation. Studies at the population level (*n* = 8) relied on national survey datasets or administrative databases to analyze large samples of PWD. Rather than focusing on individual lived experiences or specific workplace contexts, these studies provided macro-level insights into broad patterns of inequality and structural influences on employment outcomes. Such perspectives are valuable for identifying population-level disparities and informing policy development (14, 46).

This review synthesizes evidence across individual, organizational, and population levels, showing how different types of clients contribute unique yet complementary perspectives. Individual-level studies illuminate the nuanced realities of individual lived experiences, organization-level studies highlight the systemic and programmatic supports that shape work participation, and population-level studies reveal broad demographic and policy-related patterns. This multi-level evidence offers a comprehensive understanding of the multifactorial influences on employment for PWD.

### 4.2 Person

The Person domain includes the intrinsic factors of an individual, such as cognitive, psychological, physiological, sensory, motor, and spiritual traits, along with broader personal characteristics like health status, disability type and severity, education, skills, prior work experience, and demographic or sociocultural factors (e.g., age, race or ethnicity, language proficiency, and social participation history). These factors collectively represent the individual's intrinsic capacities and personal characteristics that may support or constrain occupational performance and employment outcomes.

Cognition and practical skills emerged as a central theme. Three studies from North America reported that cognitive deficits, including poor prospective memory, significantly hinder job performance and retention (14, 38, 44). Conversely, personal attributes such as openness and assertiveness were linked to more favorable employment trajectories, including higher job satisfaction and career growth (40). In addition, several studies highlighted person-level functional capacities and learned competencies relevant to employment. In this review, within the PEOP framework, these factors are conceptualized as person-related capacities that may support or constrain occupational performance, rather than as performance outcomes in themselves. Foundational capacities, such as basic self-care and essential mobility (14), together with learned competencies such as computer literacy, driving, self-advocacy, and job-specific skills, may influence employability by shaping an individual's readiness to participate in work (14, 44). Evidence also indicates that PWD may experience disadvantages in employment-related skills compared with people without disabilities, reflecting person-level disparities in functional abilities and vocational competencies (58). These findings underscore the importance of targeted skill development in vocational rehabilitation. Motivation, self-esteem, and self-efficacy also consistently emerged as key psychological enablers of job seeking and sustained employment (40, 44, 46, 49). However, psychological barriers can equally undermine work participation. For instance, a German study found that exaggerated fears about vulnerability to injury led some PWD to avoid job seeking altogether (49). Taken together, these findings suggest that vocational rehabilitation programs should address both practical skill development and psychological readiness through interventions that enhance motivation, clarify work incentives, and strengthen career awareness (14, 42).

Biological and demographic characteristics, such as type and severity of disability, comorbidity, and age, were also repeatedly associated with employment outcomes (47, 49, 55). Employability is a core focus of rehabilitation, particularly for individuals with mental disorders (59). However, evidence indicates that those who are middle-aged or have physical disabilities tend to have higher employment prospects. For instance, a Chinese cross-sectional study reported higher employment rates among individuals aged 27–49, whereas those with intellectual, multiple, or severe disabilities faced significantly more barriers to employment (55). Evidence from low- and middle-income settings indicates intellectual disabilities are less well understood and therefore less accepted than physical disabilities (60). U.S. data suggest patterned disparities in employer hiring practices, with individuals with blindness, mental health conditions, or intellectual disabilities facing particularly low likelihoods of employment compared to other disability groups (61). Other studies identify mobility impairments, such as difficulties in walking, standing, or lifting, as particularly limiting for workforce participation (62). Co-occurring physical health conditions often further restrict employment opportunities for individuals with psychiatric disabilities (42, 46, 51), whereas improvements in general health correlate with greater employability (14). Educational attainment and prior paid work experience were consistently linked to better employment outcomes across regions in Europe, North America, and Asia (14, 49, 55). A higher degree of education can bring more employment opportunities (14, 55), and vice versa (49). Three studies conducted in North America found that prior employment experience, particularly paid work experience, was a significant positive predictor of current employment opportunities (14, 41, 45). However, one of these studies revealed that an arrest record is a substantial barrier to employment for PWD (14). In terms of race and ethnicity, in the United States, white youth with disabilities demonstrated more favorable employment outcomes compared with peers from other racial or ethnic groups (14). In contrast, employment outcomes were less favorable for Hispanic youth with disabilities (47). This disparity may, in part, reflect the historical persistence of racial discrimination in Western countries, which can influence hiring decisions, career advancement, and overall workforce inclusion (63).

Not all studies show consistent patterns: a longitudinal U.S. survey found no clear association between age or disability type and competitive employment, perhaps reflecting sample characteristics (youth populations) or the more decisive influence of transition-focused supports in those cohorts (14). Such discrepancies underscore that Person factors interact with contextual supports and that conclusions must be interpreted within study-specific settings.

### 4.3 Environment

The Environment domain refers to the physical and social contexts of employment, encompassing workplaces, transportation systems, public services, family supports, and broader policy frameworks (64, 65). These environmental conditions can either facilitate or constrain the occupational performance of PWD.

Policies shape opportunities by determining eligibility criteria and incentives. For instance, in Spain, removing tax exemptions for unemployed PWD under age 55 while retaining them for those over 55 increased younger men's employment probability by 6.5 percentage points (54). This demonstrates the significant role of well-designed public policies in promoting equitable workforce participation. Modified workspaces, such as accessible entrances, hallways, restrooms, and workstations, are essential for employees with disabilities to navigate workplaces and perform effectively (46, 64). Beyond the workplace, accessibility also extends to transportation and public facilities. Transportation has long been identified as a significant barrier: a U.S. study in 2007 highlighted that limited access to convenient transport undermines job participation for PWD (66). Recent evidence shows that transportation assistance programs can enhance employment outcomes (43, 46). Yet, findings remain mixed. A longitudinal U.S. study reported that local public transportation was not significantly associated with competitive employment, possibly because the youth participants relied more on driving themselves; indeed, possessing a driver's license positively predicted successful employment (14).

Workplace culture significantly impacts employment outcomes. Negative working experiences, such as abusive behaviors or strained interpersonal relationships, can create significant barriers to job retention (67). In some Asian contexts, such as Singapore, workplace cultures shaped by concerns about employees' behavior or the Organization's reputation may contribute to negative perceptions of workers with mental health conditions (68). In low- and middle-income countries, employer misconceptions about the capabilities of individuals with intellectual disabilities further limit opportunities (60). More broadly, the interplay of highly competitive labor markets and societal biases against PWD restricts their access to work, affecting both employers' hiring decisions and employees' career advancement prospects (41, 42, 46). Conversely, workplaces that value diversity and inclusion can mitigate attitudinal barriers and foster better recruitment, retention, and career progression for PWD (17, 69, 70). For example, supportive attitudes and assistance from colleagues and supervisors improve workplace adjustment for PWD (43, 46). Employers who hold open-minded and inclusive views are more likely to provide opportunities (40, 43), while negative or indifferent attitudes remain a significant barrier (46). Employer awareness and provision of assistive technologies can substantially enhance workplace integration. Analysis of U.S. Current Population Survey data (2012–2021) found that assistive technologies mitigate functional limitations and improves employment outcomes for PWD (62). Technologies like screen readers, voice recognition software, and ergonomic workstations increase independence, productivity, and job accessibility (71). Satisfaction with assistive technologies use is generally high, particularly among people with blindness or low vision (72). Still, barriers such as cost and inadequate support systems restrict widespread adoption.

Families provide another critical dimension of environmental influence. Having a parent or guardian in the household positively predicted employment for PWD in U.S. samples (14). Socioeconomic background also matters: families with higher resources often provide better access to education, networks, and emotional support, enhancing employment prospects. Higher parental expectations and encouragement can strengthen confidence and self-esteem, helping PWD navigate the complexities of employment (41). Conversely, overprotection or low expectations may hinder independence and career development. Furthermore, caregiving responsibilities like childcare can divert PWD from seeking or sustaining employment (46).

### 4.4 Occupation

The Occupation domain focuses on the roles, tasks, and activities through which PWD engage in employment. Vocational rehabilitation interventions and training programs have substantially improved employment outcomes (39, 40, 46, 50, 52, 53). Supported employment programs have proven particularly valuable, offering structured assistance that enables workers with disabilities to secure and sustain competitive jobs (47, 73). On-site job instructions, mentorship from return-to-work coordinators, and job coaching provide individualized guidance that helps employees with disabilities adapt to their roles (46, 74–76). Employers also contribute by offering remote work options, flexible schedules, and part-time opportunities, which accommodate diverse needs and foster greater workforce participation (37, 77). Conversely, heavy or overly complex workloads can undermine employment sustainability (42). Simplified job tasks and tailored work schedules can mitigate these challenges and promote better outcomes (37, 46).

Interestingly, engagement in physical or recreational activities has been linked to improved employment outcomes. A U.S. study of 3,076 participants found that engagement in physical activity was associated with an 8.4% higher likelihood of employment, highlighting the role of health-promotion behaviors in supporting work participation for PWD (78). Another U.S. study of 606 participants found that individuals participating in adaptive video gaming exhibited higher employment rates than the general PWD population (48). From their research, adaptive gaming enhances mental health, physical function, and cognitive abilities, supporting workplace adaptability and productivity. Thus, integrating such recreational and physical activities into vocational rehabilitation programs may provide novel avenues for employment preparation, retention, and career advancement. In the future, such interventions can be tailored to the specific demands of the job, using physical and recreational activities designed to develop the broader skills and capacities that support sustained workforce participation.

### 4.5 Implications for occupational therapy

Using the fourth edition of the PEOP model, this review identified person-, environment-, and occupation-related factors that shape employment outcomes for PWD and highlighted their implications for occupational therapy practice and research. The PEOP model offers occupational therapy practitioners a distinctive lens by framing employment not merely as an individual outcome, but as a form of occupational performance shaped by the interaction among personal capacities, environmental supports and barriers, occupational demands, and the client's lived context. This perspective is particularly valuable in vocational rehabilitation because it supports a more comprehensive and client-centered understanding of how work participation may be facilitated or constrained.

Across the included studies, the influence of similar factors on employment outcomes varied according to demographic characteristics, cultural contexts, and employment environments. Such variation suggests that clients may differ in their perceptions, needs, goals, and lived experiences of work. Therefore, meaningful vocational rehabilitation practice should begin with careful consideration of the client type and narrative (18). For occupational therapy practice, these findings suggest that employment issues should be assessed across all three PEOP domains. Taken together, the findings reinforce the role of occupational therapists in conducting occupation-based and narrative-informed vocational assessments, identifying modifiable barriers, matching individuals to suitable work roles, and collaborating with employers, families, and service systems to support sustainable employment outcomes. In this way, occupational therapists can tailor interventions and recommendations to specific job contexts, thereby enhancing their practical relevance and effectiveness in supporting employment among PWD.

To illustrate how these implications may be applied in a context-specific manner, the sewing industry in mainland China provides a useful example. This sector is experiencing a significant labor shortage, with demand for workers exceeding the available supply of job seekers (79). Sewing work may represent a realistic employment option for some PWD because it often involves relatively lower physical demands and may therefore be more accessible to individuals with reduced physical capacity (80). In this context, understanding the client's narrative is an important starting point, because employment experiences, goals, and perceived barriers may differ across individuals, workplaces, and broader populations. For instance, individual narratives vary among sewing workers, necessitating firsthand interviews to understand their unique experiences. At the organizational level, employment dynamics differ across textile factories, necessitating site visits to capture workplace-specific factors. At the population level, perspectives of people with different types of disabilities or from other countries should be aggregated and analyzed separately. Once the client's narrative has been understood, the PEOP model can be used to examine how person, environment, and occupation factors may influence successful employment. Within the PEOP model, person factors may influence sewing employment through motivation, self-efficacy, health status, functional abilities, prior work experience, and vocational skills, all of which may affect whether a worker can learn, perform, and sustain sewing tasks. Environmental factors may include workplace accessibility, employer attitudes, coworker support, transportation, family support, and the broader organizational climate, which together shape opportunities for workplace inclusion and retention. Occupational factors may include the specific task demands of sewing work, workload, work pace, task structure, flexibility of work arrangements, and the availability of accommodations or job modifications. Systematically examining these domains can help occupational therapy practitioners identify where barriers arise and what forms of support are needed. In turn, this may enhance occupational performance, job retention, and sustained employment outcomes for PWD in China's sewing industry.

In the context of the rapid development of artificial intelligence, future occupational therapy research may also explore whether data-driven approaches, such as predictive modelling, can complement PEOP-informed vocational assessment by identifying combinations of factors associated with stable employment outcomes. For instance, a study on cardiac rehabilitation evaluated eight machine-learning models and identified key factors influencing return to work, such as driving ability, heart rate, and age (81). Another study used elastic net logistic regression, random forest, and gradient boosting to predict the return-to-work outcome after traumatic brain injury and identified pre-injury employment status as the most significant predictor (82). By collecting and analyzing data on the demographic characteristics of PWD and relevant personal, environmental, and occupational factors, researchers could use machine learning techniques to assess the importance of these factors to employment outcomes for clients with different narratives (83). Such an approach may help identify key predictors of stable employment across job roles and provide more precise, data-driven recommendations for job matching. Ultimately, this line of research could strengthen the evidence base for occupational therapy and support more contextually responsive vocational rehabilitation interventions for PWD.

### 4.6 Limitations and future research

This study has several limitations. First, the included studies did not contribute equally to the synthesis, and the inclusion of diverse disability groups requires caution when interpreting and generalizing the findings. Second, methodological variation across studies limited meaningful comparison of results. Third, publication bias may have influenced the evidence base by increasing the visibility of studies reporting positive findings.

Despite these limitations, the review highlights several directions for future occupational therapy research. More longitudinal studies are needed to examine how person-, environment-, and occupation-related factors influence not only job acquisition, but also long-term job retention, work performance, and career development. Intervention studies are also needed to evaluate which combinations of person-focused, environment-focused, and occupation-focused strategies are most effective for different disability groups and employment contexts. In particular, experimental research is needed to test the real-world effectiveness of vocational training programs, assistive technologies, and employer-focused strategies. In addition, comparative and cross-cultural research is needed to examine how the meaning and impact of employment-related factors vary across sociocultural settings, service systems, and labor markets. Longitudinal and policy-relevant studies would also be valuable for identifying key transition points in employment trajectories and assessing how policy changes influence the persistence of employment barriers over time. Notably, this review included only two developing countries, China and Greece, underscoring the need to expand research to more diverse geographic regions, particularly low- and middle-income countries, in order to better understand global disparities and develop culturally responsive interventions.

## 5 Conclusion

This scoping review applied the latest edition of the PEOP model to synthesize evidence from studies published between 2015 and 2025. By examining client types and categorizing influencing factors within the Person, Environment, and Occupation domains, the review mapped how these dimensions interact to shape employment for PWD. This framework highlights the complex and dynamic interplay across client levels and PEOP domains, advancing academic understanding of the multifaceted nature of disability employment. The findings also provide practical guidance for policymakers, employers, and vocational rehabilitation practitioners in promoting equitable workforce participation for PWD.

## Data Availability

The original contributions presented in the study are included in the article/Supplementary Material, further inquiries can be directed to the corresponding authors.

## Supplementary material

Supplement Table 1 Search strategy.

Supplement Table 2 General characteristics of the studies.
