# Supplementary material for: Exploring factors shaping employment outcomes of people with disabilities through the PEOP model: a scoping review
Source: Front Rehabil Sci. 2026 Apr 28;7:1725152. doi: 10.3389/fresc.2026.1725152 (PMC13162215; doi:10.3389/fresc.2026.1725152)
Supplement: Supplement Table 1 — Search strategy. [file Table1.docx]

Search strategy

| Database | Number | Search strategy |
| --- | --- | --- |
| CNKI (China National Knowledge Infrastructure; Chinese: 中国知网) | n1=799; n2=77 | TKA=残疾人 AND TKA=就业 研究论文 |
| Pubscholar (Chinese: 公益学术平台) | n1=1263; n2=139 | TS=就业 AND TS=残疾人 近十年 期刊论文 |
| Pubmed | N1=1472; n2=35 | ("Employment"[Mesh] OR "Work"[Mesh] OR "Occupations"[Mesh] OR "Career Choice"[Mesh] OR "Job Security"[Mesh] OR "job"[Title/Abstract]) AND ("Disabled Persons"[Mesh] OR "people with disabilities"[Title/Abstract] OR "disabled people"[Title/Abstract] OR "disabled persons"[Title/Abstract] OR "persons with disabilities"[Title/Abstract]) AND ((y_10[Filter]) AND (humans[Filter])) Filters: in the last 10 years, Humans, English |
| Embase | n1=2143; n2=312 | ('employment'/exp OR 'work'/exp OR 'occupation'/exp OR 'job security'/exp OR 'job':ti,ab,kw) AND ('disabled person'/exp OR 'people with disabilities':ti,ab,kw OR 'disabled people':ti,ab,kw OR 'disabled persons':ti,ab,kw OR 'persons with disabilities':ti,ab,kw) AND [english]/lim AND [2015-2025]/py AND [article]/lim |
