# Supplementary material for: Exploring factors shaping employment outcomes of people with disabilities through the PEOP model: a scoping review
Source: Front Rehabil Sci. 2026 Apr 28;7:1725152. doi: 10.3389/fresc.2026.1725152 (PMC13162215; doi:10.3389/fresc.2026.1725152)
Supplement: Supplement Table 2 — General characteristics of the studies. [file Table2.docx]

**General characteristics of the studies**

| **Evidence Table for Factors Affecting the Employment of People with Disabilities** | | | | | |
| --- | --- | --- | --- | --- | --- |
| Author/Year | Study Design | Participants  Inclusion Criteria  Study Setting | Intervention and Control Groups | Outcome Measures/Data collection | Results |
| **Qualitative Studies** | | | | | |
| Bal et al., 2017 | Qualitative (semi-structure interviews) | *Participants (Individual)*  19 young adults with chronic physical disabilities (age range: 16-27yr, 42.1% male)  *Inclusion Criteria*  Persons enrolled in the “At Work” intervention program indicated their interest in participation  *Study Setting*  Outpatient rehabilitation center and vocational reintegration company. | N/A | Outcomes were measured through semi-structured interviews with 19 former participants.  The interviews explored:  Perceived barriers and facilitators for employment.  Beneficial attributes of the program.  Recommendations for additional components. | *Significant Findings* ***(Person, Environment, Occupation)***  Facilitators: employers’ attitude (supervisor's attitude: positive, open-minded and showing understanding), self-esteem and self-efficacy, vocational training programs (“At Work” program)  Barriers: basic daily living ability (limited physical functions and capacities), personal character factors (not being open and assertive)  *Nonsignificant Findings*  None |
| Lindsay et al., 2019 | Descriptive qualitative design (in-depth interviews) | *Participants (Individual)*  The study involved a purposive sample of 33 participants: 23 youth with physical disabilities (13 females with a mean age of 22.9yr and 10 males with a mean age of 21.3yr), and 10 clinicians who assist youth in their transition to adulthood and employment.  *Inclusion criteria*  Youth: Aged 18-25 with physical disabilities, recruited through a large urban pediatric rehabilitation hospital in Ontario, Canada. Participants had to be involved in at least one appointment with a clinician offering post-secondary transition services from 2004-2015. Clinicians: Professionals with at least one year of paid experience helping youth transition to adulthood, currently or recently working with youth with disabilities.  *Study setting*  A large urban pediatric rehabilitation hospital in Ontario, Canada. | N/A | Person Level: Managing their condition, self-advocacy, and willingness to ask for help.  Environment Level: Parental and social support, accommodations, stigma and discrimination, and transportation challenges.  Occupation Level: Different levels of engagement in employment, with males and females showing different patterns of career. | *Significant Findings* ***(Person, Environment)***  Facilitators: advanced skills (ability to manage their condition, self-advocacy), character factors (willingness to ask for help), parental support, social support, accommodations, work experiences, social activities participation (volunteer experiences)  Barriers: stigma and discrimination, transportation challenges, parental expectations (lowered expectations and overprotection from parents)  *Nonsignificant Finding*  None |
| Vlachou et al., 2021 | Qualitative (Semi-structured interview) | *Participants (Individual)*  14 participants (8 people with intellectual disabilities and 5 people with mental health conditions; 8 male and 6 female; mean age: 34.4yr, age range: 26-50yr)  *Inclusion criteria*  Participants had to have at least one job placement during their cooperation with the supported employment agency.  *Study Setting*  Three supported employment agencies in Greece. | N/A | Primary Outcome: The primary outcomes measured were the experiences and perceptions of the participants regarding the facilitators and challenges in finding and maintaining a job in the open labor market during their participation in supported employment programs. | *Significant Finding* ***(Person, Environment, Occupation)***  Facilitators: supported employment services (direct mediation of the supported employment professionals; directions and guidance; assistance in decision-making regarding employment Issues; the exploration of work’s personal incentives and working preferences; emotional benefits; acquisition of skills; assistance in practicalities; interaction and support provision in the workplace)  Barriers: labor market challenges (limited employment opportunities, competition and/or an excessive number of prospective employees), comorbidity factors (the implications of chronic health conditions); inclusion (difficult and stressful inclusion employment sectors, adaptation process to the new job environment created confusion and anxiety; adjustment period was overwhelming), interpersonal challenges (difficulties in the collaboration and relations with their colleagues), stigma and discrimination, (abusive behaviors in the workplace), workload (heavy workload leading to fatigue)  *Nonsignificant Finding*  None |
| Ponzio, et al, 2024 | Qualitative Study based on two focus groups | *Participants (Individual)*  10 females with multiple sclerosis reporting disability.  *Inclusion Criteria*  The participants were identified through the local sections of the Italian MS Association (AISM) based on the following selection criteria: male or female; being of working age (18-65 yr); either being in work or out of work for no more than 3 years.  *Study Setting*  Web conferencing in Italy | N/A | Interview’s analysis was guided by a (constructivist) grounded theory.  Topics that emerged during the focus group sessions within the different areas of discussion including Aspects influencing job retention. | *Significant Finding* ***(Environment)***  Facilitators: role of colleagues, employers’ support (the presence of support for managing work-life demands).  Barriers: transportation (the burden of getting to and from work.).  *Nonsignificant Finding*  None |
| **RCT** | | | | | |
| Burton et al., 2019 | RCT | *Participants (Organization)*  58 participants were diagnosed with schizophrenia/ schizoaffective disorder, 37 were diagnosed with bipolar disorder, and 58 were diagnosed with major depressive disorder. (mean age 44yr, 57% male)  *Inclusion Criteria*  (a) unemployed for at least 30 days, (b) DSM-IV diagnosis of schizophrenia, schizoaffective disorder, bipolar disorder, or major depressive disorder, and (c) unemployed and stating a current goal of employment.  *Study Setting*  outpatient psychiatric care at a university clinic | *Intervention group:* supported employment with Compensatory Cognitive Training  *Control group:*  supported employment without Compensatory Cognitive Training | Real-world functional status included work history variables, clinical history variables, baseline functional capacity (UCSD Performance-based Skills Assessment-Brief), and work outcomes (weeks worked and wages earned during two years of supported employment). | *Significant Findings* ***(Person)***  Barriers: Cognition and memory (“…worse prospective memory performance was associated with ... worse employment outcome measured prospectively.”)  *Nonsignificant Findings*  “MIST performance did not significantly predict variance in work in the past five years” |
| Zhang et al., 2017 | RCT | *Participants (Organization)*  162 participants with schizophrenia (3 groups of mean age: 32yr, 34yr, and 31yr; males 44.4%, 38.9%, and 42.6%)  *Inclusion Criteria*  18yr; Diagnosed with schizophrenia for at least 2 years; Unemployed; No obvious cognitive, learning, or neurological impairment; Completion of primary school education; A desire to work.  *Study Setting*  Wuxi Mental Health Center, Wuxi Tongren International Rehabilitation Hospital, Nanjing Medical University, Nanjing, China. | *Intervention Group:* Participants in the Integrated Supported Employment (ISE) program.  *Control Groups:*  IPS Group: Participants in the Individual Placement and Support (IPS) program.  TVR Group: Participants in the Traditional Vocational Rehabilitation (TVR) program. | Employment Status: Assessed the number of job interviews attended, jobs obtained, hours per week worked, and salary received from each job.  Job Tenure: Measured the longest job duration sustained by each participant during the study.  Psychiatric Status: Assessed using the Brief Psychiatric Rating Scale (BPRS).  Self-Efficacy: Assessed using the Chinese General Self-Efficacy Scale (CGSS).  Psychosocial Functioning: Assessed using the Global Assessment of Functioning (GAF).  Quality of Life: Assessed using the Personal Wellbeing Index (PWI).  Job Termination: The number of unwanted job terminations per participant | *Significant Findings* ***(Occupation)***  Facilitators: supported employment (Integrated Supported Employment (ISE) program within work-related social skills training (WSST))  *Nonsignificant Finding*  None |
| **Cohort Studies** | | | | | |
| Corbière et al., 2017 | Prospective cohort study | *Participants (Organization)*  489 persons with a severe mental illness (mean: 39yr, 53.6% male) and 97 employment specialists  *Inclusion Criteria*  Participants more than 18yr presenting with a mental disorder, who were looking for a job and who were newly enrolled in a Support Employment (SE) program were eligible to participate in the study.  *Study Setting*  Across three Canadian provinces (British Columbia, Ontario, and Quebec) from 2006 to 2013 involving 24 SE programs | N/A | Competitive Employment: The primary outcome is whether participants obtained competitive employment during the study. Competitive employment is defined as work in the competitive labor market that is performed on a full-time or part-time basis in an integrated setting and for which individuals are compensated at or above the minimum wage​ | *Significant Findings* ***(Person, Environment, Occupation)***  Facilitators: age (younger age), work experience (shorter duration of unemployment), motivation (to find a job), job search strategies (active and preparatory), working alliance (with the employment specialist), interpersonal relationships (relationships with employers and supervisors), self-esteem (as a worker)  Barriers: Cognition (cognitive deficit), integration, self-efficacy (perceived barriers to employment by a scale assessing potential barriers to work integration and self-efficacy)  *Non**significant Findings*  Clinical variables (psychiatric diagnosis, substance misuse, and severity of symptoms) |
| Echarti et al., 2020 | Retrospective, quasi-experimental, cohort study | *Participants (Population)*  2399 individuals with complete employment records from three groups (mean age 41yr female 33%, mean age 37yr female 37%, mean age 38yr female 31%)  *Inclusion Criteria*  The study population consisted of people with disabilities who, due to their health problem, were no longer able to (or were it was predicted that they will in the foreseeable future no longer be able to) carry out prior job tasks. Additionally, they must have successfully applied for vocational rehabilitation with the German Statutory Pension Insurance Fund.  *Study Setting*  The data used for this analysis was retrieved from administrative records made available by the Research Data Centre of the German Statutory Pension Insurance Fund. | An internal comparison group design was used to compare outcomes with and without completed vocational re-training.  *Control group:* who were admitted into a re-training program but never received  the full benefit from training (due to no-show or dropout) (n=367).  *Intervention group:* individuals who had completed either a 1-year vocational re-training program (n=278), or a 2-year vocational re-training program (n=1754) | The impact of the re-training measures was assessed by estimating average treatment effects of graduating from a re-training program in comparison to the scenario of unsuccessful program completion (program dropout). Participation in paid employment was assessed using nominal and inflation-adjusted income, as well as employment days, in the first 8 years after program admission. Secondary outcomes comprised days on social-security benefits, days on short-term unemployment benefits, days on long-term unemployment benefits and an indicator variable signaling whether a pension due to a reduced earnings capacity was awarded. | *Significant Finding* ***(Occupation)***  vocational training programs (Both types of vocational re-training programs analyzed significantly improved the income and employment situation of the individuals involved over the first 8 years after program admission.)  Facilitators: vocational training programs (1-year and 2-year vocational re-training)  *Non**significant Finding*  None. |
| Kaehne, 2016 | Observational program evaluation study | *Participants (Organization)*  315 young people with intellectual disabilities (age range: 16-27yr, male 61.9%)  *Inclusion Criteria*  Participants of Project SEARCH of Young people with intellectual disabilities who are about to leave school or college*.*  *Study setting*  The Program Specialist Europe facilitated contact with all operational UK sites (n = 17) | N/A | The main outcome measured was employment status, defined as obtaining competitive employment in an integrated setting, with employment rates and the nature of employment (full-time or part-time) being key metrics. Full-time employment was defined as paid work for more than 16 hours per week, and part-time work was considered up to 16 hours per week. | *Significant Finding* ***(Occupation)***  Facilitator: vocational training programs (SEARCH UK)  *Nonsignificant Finding* ***(Person)***  age, disability level |
| Qian et al., 2018 | Observational Cohort Study | *Participants (Organization)*  228 students with intellectual disability (mostly with other disabilities in addition to intellectual disability) (mean age: 21yr, age range: 18–62yr, males: 50.9%)  *Inclusion Criteria*  Participants were enrolled in degree or certificate programs at these colleges and had participated in the TPSID program between 2011 and 2015.  *Study Setting*  Two community and technical colleges located in rural Midwestern communities. | N/A | Paid employment status at or above minimum wage during the most recent year in the Transition and Postsecondary Programs for Students With Intellectual Disabilities (TPSID) program. | *Significant Finding* ***(Person)***  Facilitators: work experience (prior paid work experience), social activities participation (attended organized social events, participated in volunteering and/or community service), educational experience (took only inclusive classes).  *Nonsignificant Finding*  None |
| Reims & Tisch, 2022 | Quasi-Experimental Study Using Propensity Score Matching | *Participants (Population)*  Total Number: 7,905 individuals in the first comparison group and 21,020 individuals in the second comparison group. (Gender: In the first comparison group, the treatment group: 62% males. In the control group: 55% males. In the second comparison group, the treatment group: 59% males, and the control group: 63% males. Age: The age range for the study was between 25 and 40yr.)  *Inclusion Criteria*  Individuals aged between 25yr and 40yr, who were not employed at the beginning of the vocational rehabilitation or training program.  *Study Setting*  German Federal Employment Agency | *Intervention Group:* Rehabilitants participating in vocational training programs. This included both general and disability-specific training programs.  *Control Group:*  In the first comparison, rehabilitants who did not participate in any program. In the second comparison, non-rehabilitants who had health conditions and were participating in the same general training programs. | Employment Outcomes: Unsubsidized employment, marginal employment, and subsidized employment.  Income: Mean yearly income from paid work and social benefits.  Employment Duration: Number of days worked per year.  Lock-in Effect: During the training period, participants were less likely to find employment, showing a lock-in effect. However, positive effects were observed after completing the training programs, with higher chances of unsubsidized employment and longer employment durations.  Income Effects: No significant income difference after four years, but initially, non-participants had higher income levels. | *Significant Finding* ***(Occupation)***  Facilitators: vocational training programs (Training participation helps to improve employment participation of rehabilitants. Vocational rehabilitation status is associated with positive employment and income opportunities.)  *Nonsignificant Finding*  None |
| Vall, 2017 | Quasi-Experimental Study Using Difference-in-Difference and Triple Difference Models | *Participants (Population)*  Male, younger than 65yr.  Broad Control Group: 12,511 individuals in the sample, resulting in 65,887 person-year observations.  Restricted Control Group: 974 individuals in the sample, resulting in 5,010 person-year observations.  *Inclusion Criteria*  Partial Disability: Individuals classified as partially disabled according to the Spanish Social Security Administration.  Age: Primarily those aged 55yr or younger for the treatment group, with those older than 55yr used for additional comparison.  Residence: Living in the province of Bizkaia for the treatment group, with control groups from other provinces in Spain.  *Study Setting*  Continuous Sample of Working Lives (MCVL), a microeconomic dataset from the Spanish Social Security Administration | *Intervention Group:*  Description: Partially disabled individuals in Bizkaia aged 55 or younger, who were affected by the policy change eliminating tax exemptions if they did not work.  *Control Group:*  Description: Two control groups were used: (1) all other provinces in Spain except Guipuzkoa and (2) only the five neighboring provinces of Bizkaia (Alava, Cantabria, Navarra, La Rioja, and Burgos). | The primary outcome measured was the employment status of disabled individuals, particularly the probability of working. This was assessed using a difference-in-difference model and a triple difference model. | *Significant Finding* ***(Environment)***  Facilitators: reform of policy and laws (A reform in Spain in 2007).  *Nonsignificant Finding*  None |
| Wehman et al., 2015 | Longitudinal Survey Design | *Participants (Population)*  2,900 students with disability (mean age 16yr)  *Inclusion criteria*  Youth with disabilities who were aged 16yr at the beginning of the 2000–2001 school year and who had left high school by the 2003–2004 school year.  Study setting  United States. The study utilized data from the National Longitudinal Transition Study-2 (NLTS-2). | N/A | Competitive employment, defined as any paid job where the youth made at least minimum wage and was employed in a setting where most employees did not have disabilities. | *Significant Finding* ***(Person, Environment, Occupation)***  Facilitators: youth’s race/ethnicity (white), education experience (college level, General Educational Development Certificate, school characteristics: regular high school, high school graduation, better classroom social score and classroom behavior score, student better attendance at Individualized Education Program (IEP) meetings, participating in either a post-secondary vocational school or 4-year college/university program, or any post-secondary school program), social activities participation (active participation in extracurricular activities, participation in community activities), basic ability of daily living (self-feeding, self-dressing, ability to get to places), cognition (youth’s ability to understand), work experience (having had a job while still in high school), general health (general health of the youth), family economy (high level income, families receiving no benefits), household, parental expectation (higher parental expectation of both a paid job and self-support), advanced skills (higher levels of communication, high level of self-advocacy, computer skills, having a driver’s license), career awareness training, native English speakers (being a native English speaker)  Barriers: having an arrest record  *Nonsignificant Finding*  heavy smoking or drinking, gang affiliation, and use of illegal substances, type of disability, gender of the youth, community type, living situation, age, general, public transportation, student’s grades, the parent’s attendance in the IEP meetings, the youth’s role in the IEP meetings, and the engagement and enjoyment of the student |
| **Cross-Sectional Studies** | | | | | |
| Emerson et al., 2018 | Cross-Sectional Analysis of a Cohort Study | *Participants (Population)*  The study analyzed data from the 1970 British Cohort Study (BCS70), which includes over 17,000 children born during one week in the UK in 1970.  *Inclusion Criteria*  British adults with and without intellectual impairments at ages 26yr (n = 8654), 30yr (n = 10,833), 34yr (n = 9316), 38yr (n = 8874) and 42yr (n = 9717).  *Study setting*  BCS70, a nationally representative data from eight waves of the 1970 British Cohort Study managed by Centre for Longitudinal Studies at University College London and is funded by the UK’s Economic and Social Research Council. | N/A | **Self-Rated Health: The study examined self-rated health among participants, categorized as 'poor' or 'fair' and others.**  **Mental Health: The presence of potential mental health problems was assessed.**  **Economic Activity: Employment status was measured, including full-time employment, part-time employment, and unemployment or economic inactivity.**  **Health Outcomes and Economic Activity: The study explored the prevalence of poor health in relation to economic activity status, such as unemployment, education/training, and ill/disabled status.** | *Significant Finding* ***(Person)***  Barriers: general health (poorer self-rated health and mental health)  *Nonsignificant Finding*  None. |
| Paul & Hollederer, 2023 | Cross-Sectional Study | *Participants (Population)*  739 people with disabilities (mean age: 51.4yr, age range: 16–65yr, female: 37.5%)  *Inclusion Criteria*  Participants were included if they were part of the labor market, either employed or officially registered as unemployed.  *Study Setting*  Location: The study was conducted in Germany, using data from the Panel Arbeitsmarkt und Soziale Sicherung (PASS), which is maintained by the Research Data Center at the Institute for Employment Research of the Federal Employment Agency. | N/A | Employment Status: Measured as either employed or unemployed.  Job Search Behavior: Analyzed through logistic regressions, focusing on seven different methods of job search and the reasons for not searching for a job among those unemployed.  COVID-19-Related Worries: Measured using three Likert items querying participants' worries about their own health, the health of their family, and their financial situation during the pandemic. | *Significant Finding* ***(Person, Environment)***  Barriers: disability type (cardiovascular diseases, mental illnesses, and musculoskeletal disorder); education experience (a low level of formal education: not more than secondary school certificate), advanced skills (a lack of professional qualifications), place of birth (not domestic place of birth), age (lower age: younger than 50), household (a small number of children in the household), disability severity (severe), motivation (reluctance to work because of health-related considerations or unsatisfied financial situation)  *Nonsignificant Finding*  None |
| Sevak & Khan, 2017 | Observational Cross-Sectional Study | *Participants (Population)*  Total: 2,148 individuals, Age: 25-60yr (1,309 individuals with physical disabilities only 48.9% male, 440 individuals with psychiatric disabilities only 52.4% male, and 399 with both physical and psychiatric disabilities, 46.4% male.)  *Inclusion Criteria* Individuals aged 25 to 60 who applied for vocational rehabilitation (VR) services  *Study Setting*  Individuals who applied for VR services in 2014 in agencies in Mississippi, New Jersey, and Ohio. | N/A | Employment Status: Measures included employment history, current employment status, and reasons for not being employed.  Barriers to Employment: Factors such as health, being fired, transportation issues, and lack of skills were examined.  Facilitators to Employment: Receipt of workplace accommodations, such as job coaching, flexible schedules, and modified work duties, were analyzed to determine their impact on employment outcomes. | *Significant Finding* ***(Person, Environment, Occupation)***  Facilitators: vocational training programs (job coach and training), counseling (counseling on work benefit, counseling and therapy), transportation (help with transportation), flexible work schedules, modified job duties, workplace accessibilities (accessible modified workspace), assistance from coworkers, caregiving issue (assistance in caregiving, help with child and family care, personal care and assistant)  Barriers: general health (health challenges), employers’ attitude (negative, fired, employers won’t give them a chance), caregiving issue (childcare or personal issue), advanced skills (lack of vocational skills), transportation (lack of reliable transportation), motivation (lack of motivation, did not like or need, discourage from previous attempt, not want to lose SSDI/Medicaid), workplace accessibilities (workplace not accessible), labor market challenges (cannot find job)  *Nonsignificant Finding*  None |
| Wong et al., 2022 | Cross-sectional design | *Participants (Organization)*  733 people with physical disabilities (female: 54.9%, mean age: 49yr), 67 employers.  *Inclusion Criteria*  People with disabilities: At least 18yr; Ability to speak and understand English; Having a physical disability.  Employer:  Age 18 yr and older; Ability to speak and understand English; Involvement in hiring, accommodations, or managing employees.  *Study Setting*  Location: Midwestern United States, primarily within the state of Illinois. | N/A | Employment Effects: Assessed the impact of the COVID-19 pandemic on the employment status of PWD and the business changes experienced by employers.  Challenges During Pandemic: Examined the challenges PWD faced during the pandemic, including employment changes, financial issues, and health risks.  Employer Responses: Evaluated how employers adapted to the pandemic, including changes to business operations, remote work, and employee accommodations. | *Significant Finding* ***(Person)***  Barriers: Marital status (single), race/ethnicity (Hispanic), age (younger)  *Nonsignificant Finding*  None |
| Wu, 2018 | Cross-Sectional Study | *Participants (Organization)*  115 people with disabilities in Fuzhou (55.7%: males, age range: 16-60yr)  *Inclusion Criteria*  Participants included individuals with disabilities living in Quanzhou City who were of working age and had disability.  *Study Setting*  Quanzhou City, Fujian Province, China. | N/A | Employment Status: This includes the number of employed, semi-employed (temporary or informal work), and unemployed individuals among the participants.  Income Level: The average monthly income of disabled individuals compared to the average monthly income of the general population in Quanzhou. | *Significant Finding* ***(Person, Environment)***  Facilitators: age (middle age 27-49y), education experience (higher education level), labor ability, social support (by Social Support Scale including subjective support: emotional experiences of respect and understanding; objective support: tangible assistance and social networks.)  Barriers: disability type (mental, intellectual, multiple), disability severity (sever)  *Nonsignificant Finding*  None |
| Redepenning et al., 2024 | Cross-sectional study survey | *Participants (Organization)*  606 participants with physical disabilities  *Inclusion Criteria*  must be 18 yr or older; must have a physical or neurologic condition preventing the use of a standard game controller; must be currently using adaptive gaming equipment or modifications for video gaming; must have no less than six months of experience using adaptive gaming equipment or modifications.  *Study Setting*  Multiple rehabilitation sites, national rehabilitation organizations, and adaptive gaming organizations in the United States. | N/A | The survey consisted of multiple sections, which gathered the following information: Sociodemographic data: age, gender, race, ethnicity; Highest level of education; Veteran status; Primary diagnosis; Gaming habits; Current employment status; Occupation; Frequency of use of gaming equipment to complete tasks related to employment. | *Significant Finding* ***(Person, Occupation)***  Facilitators:  Adaptive Gaming (Employment rates in individuals with disabilities who participate in adaptive gaming would be higher than employment rates of individuals with disabilities in the general population)  Education level (above high school); Identity (Veteran status).  *Nonsignificant Finding*  Use of adaptive gaming equipment for other activities (excluding game) |
| Jessiman-Perreault et al., 2025 | A Cross-Sectional Analysis | *Participants (Population)*  955 Canadians working with physical and/or mental/cognitive disabilities  *Inclusion Criteria*  18 yr or older; currently employed or employed for at least 3 months in the previous year; working at least 12 hours per week.  *Study Setting*  A survey research firm with an existing panel (ie, probability-based public opinion research panel) of over 90,000 Canadians assembled using random digit dialing and designed to mirror the population in Canada | N/A | Variables: Job Disruptions; Workplace Accommodations  Potential confounding variables: (1) individual factors (disability type, sex, age, education, caregiving responsibilities), (2) work context factors (union membership, shift work, work hours, workplace size), and (3) workplace perceptions (job insecurity, temporariness of work, vulnerability of work) | *Significant Finding* ***(Environment, Occupation)***  Facilitators:  workplace accommodations and modifications; work schedule flexibility  *Nonsignificant Finding*  None |
| *Note.* N/A= Not Applicable, yr = years old, RCT = Randomized Controlled Trial | | | | | |
